# Longitudinal evaluation of RADUCATION: a digital learning environment for the radiology residency structured to a competency-based curriculum by the German Young Radiology Forum

**DOI:** 10.1186/s13244-025-02135-x

**Published:** 2025-12-08

**Authors:** Isabel Molwitz, Katharina Stahlmann, Manuel Kolb, Marian Feiler, Nadine Bayerl, Marc Kuennemann, Fiona Mankertz, Inka Ristow, Gerhard Adam, Barbara Daria Wichtmann, Robert Rischen, Anne Frisch

**Affiliations:** 1 Department of Diagnostic and Interventional Radiology and Nuclear Medicine, University Medical Center Hamburg-Eppendorf, Hamburg, Germany; 2 Institute of Medical Biometry and Epidemiology, University Medical Center Hamburg-Eppendorf, Hamburg, Germany; 3 Department of Radiology, Health NZ - Te Whatu Ora Waikato, Hamilton, New Zealand; 4 urbanstudio GmbH, Berlin, Germany; 5 Institute of Radiology, Universitätsklinikum Erlangen, Friedrich-Alexander-Universität Erlangen-Nürnberg, Erlangen, Germany; 6 Clinic for Radiology, University Hospital Muenster, Muenster, Germany; 7 Department of Diagnostic and Interventional Radiology, Eberhard Karls University Tuebingen, Tuebingen, Germany; 8 Clinic of Neuroradiology, University Hospital Bonn, Bonn, Germany; 9 German Center for Neurodegenerative Diseases (DZNE), Bonn, Germany; 10 Department of Diagnostic and Interventional Radiology, Bielefeld University, Medical School and University Medical Center OWL, Lippe Hospital, Detmold, Germany

**Keywords:** Internship and residency, Education (medical), Training support, Curriculum, Radiology

## Abstract

**Introduction:**

RADUCATION - a digital platform for radiological postgraduate training by the German Young Radiology Forum - provides learning content structured according to the German Training Curriculum, including original board exam questions. This is the first study to evaluate user statistics, experience, and preferences.

**Materials and methods:**

Data were collected through webpage analytics and surveys. User statistics were analyzed for four periods: initial usage (05/2022–05/2023), post-introduction of theoretical board exam questions (05–11/2023), post-introduction of image-based board exam questions (12/2023–05/2024), and ongoing usage (03–12/2024). User perception surveys were conducted before (05/2023–01/2024) and after (06–08/2024) implementation of image-based board exam questions. Analyses included descriptive statistics and multivariable logistic regressions.

**Results:**

User numbers increased steadily, with board exam questions becoming the most accessed feature. Mean monthly active user numbers increased from 372 between 05/2022–05/2023 (total users 4468), to 613 between 03/2024–12/2024 (total 15,828). Survey respondents (*n* = 243) consistently rated RADUCATION as valuable for board exam preparation (88.6%), for night/weekend shifts (75.6%), and for clinical routine (73.4%). Board exam preparation was more beneficial for 4th/5th-year residents (odds ratio (OR) 1.35 (95% confidence interval (95% CI): 1.15–1.59)), while shift preparation was less critical for senior than for junior residents (OR 0.77 (95% CI: 0.59–0.99)). Video- and image-based learning content were preferred, with users rating the platform highly user-friendly (86.0%) and clearly structured (88.0%).

**Conclusions:**

RADUCATION is a valuable, widely used digital learning tool for radiology residents. Board exam questions substantially increased engagement. Its structured, peer-developed design offers a scalable model for digital postgraduate medical education across specialties and countries.

**Critical relevance statement:**

This is the first comprehensive evaluation of RADUCATION, a peer-developed, competency-based digital learning platform for the radiology residency. Integrating original board exam questions significantly increases engagement and perceived educational value, offering a scalable model for modern postgraduate medical education.

**Key points:**

- RADUCATION, a peer-developed, competency-based learning platform for radiology residents, is a highly valued, scalable model for postgraduate medical training.
- Users prefer video- and image-based content, while engagement and perceived educational value increase when integrating original board exam questions.
- RADUCATION is perceived as user-friendly and well-structured, and is considered particularly valuable for exam preparation by senior residents.

**Graphical Abstract:**

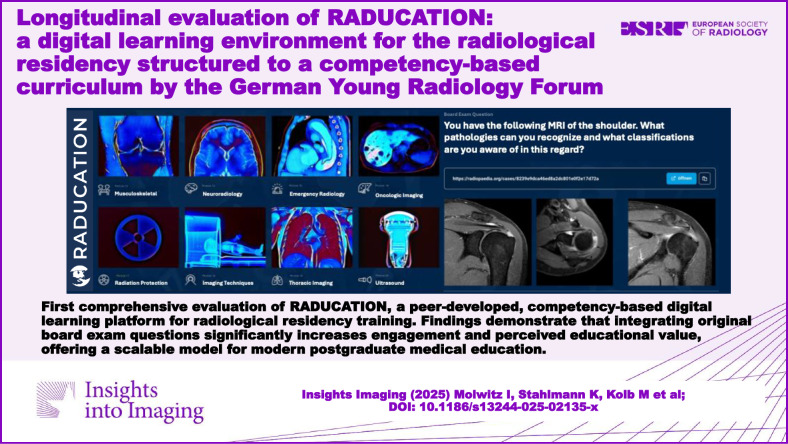

## Introduction

In 2018, the German Young Radiology Forum (Forum Junge Radiologie, FJR) of the German Roentgen Society conducted a nationwide survey about satisfaction with training conditions, including a needs assessment among radiological residents [1]. The three educational tools ranked as most effective for radiological training were supervision by a board-certified specialist (92%), a structured curriculum with a fixed rotation plan (47%), and online reference works and books (43%) [1]. Thus, the FJR developed a German Training Curriculum (GTC) to address these needs [2]. The GTC contains competency-based learning objectives based on the European Training Curriculum of the European Society of Radiology (ESR) that were adapted to national training specifics. However, learning objectives alone do not guarantee successful learning. But combined with digital teaching tools, which have become increasingly popular during the COVID-19 pandemic [3], they can support self-regulated and self-determined learning processes as effective didactic methods. Coherently, survey participants also required online learning tools [1].

Thus, RADUCATION was developed and launched in 2022. RADUCATION is a web-based tool that provides digital learning content for all GTC learning objectives. RADUCATION is structured into 21 modules that cover the chapters of the GTC, e.g., abdominal imaging, neuroradiology, pediatric radiology, radiation protection, and imaging techniques. A natural language search model enables a focused search of contents on RADUCATION. Learning objectives and the corresponding content are structured into beginner and professional levels. The beginner level is suitable for preparing for the first night and weekend shifts. The professional level is designed for board exam preparation. Care is taken to provide high-quality, free-of-charge learning content. RADUCATION enables individualized learning by creating learning lists and monitoring the learning progress. Moreover, real board exam questions from exam protocols have progressively been included since May 2023. The radiological board exam in Germany is an unstructured oral exam with theoretical and image-based questions chosen individually by the respective examiners. In Germany, 17 regional medical chambers organize these board exams. Thus, board exam questions from the protocols were assigned regionally and matched to the GTC learning objectives. On the RADUCATION website, users can therefore filter for board exam questions by their region. Board exam questions are then displayed with the corresponding learning objectives and learning contents assigned to the objectives. Additionally, best practice answers were generated based on the exam protocol and the literature in a peer-review process of FJR volunteers.

Following internationally recommended steps of curriculum development, after performing a needs assessment, creating the competency-based GTC learning objectives, and RADUCATION as an educational strategy offering learning content structured to the GTC, the next necessary step was to evaluate and improve the methods and materials used [4]. In our case, this meant evaluating the usage, perception, and value of RADUCATION to derive information on how to further improve the platform.

Therefore, this study aimed to assess user behavior and user perception concerning the RADUCATION educational web platform, with an emphasis on the impact of the implementation of board exam questions.

## Materials and methods

For this study, data on user behavior and user perceptions were obtained by (a) retrospectively available website tracking data (user statistics) and (b) by two prospectively conducted surveys among users (user experience and preferences) before and after the implementation of board exam questions in May 2023. An ethics waiver was received from the Ärztekammer Hamburg, Germany, as all data from the website tracking and both surveys were anonymized (2024-300470-WF).

### User statistics

User statistics were collected via two different web analytics systems. For data between the launch of the website in May 2022 and June 2024, Universal Analytics (Google) was employed. Since March 2024, user statistics were collected via the RADUCATION content management system (CMS) (atomic, urbanstudio GmbH), replacing Universal Analytics, which was discontinued in July 2024. Both systems measure i.a. the number of active or unique users, meaning each user is only counted once, even if she or he accesses the website several times. Also, the number of accesses on different parts of the webpage was collected. Following legal requirements, data from Universal Analytics and the CMS system were only collected upon user agreement.

**Fig. 1:**
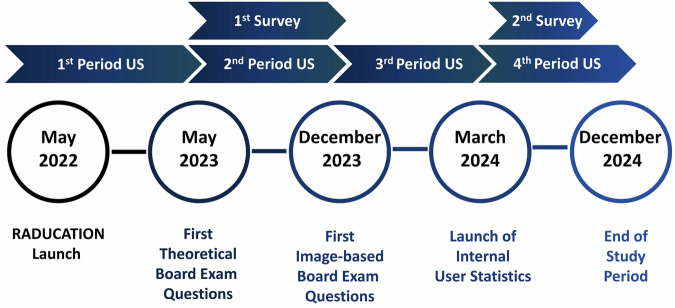
Timeline of the main developments of the RADUCATION platform, analyzed periods of the user statistics, and survey periods. The user statistics (US) were analyzed across four distinct periods, each defined by major developmental milestones of the platform, such as the introduction of the first board exam questions and the subsequent implementation of image-based questions. The first survey was conducted before implementation of image-based board exam questions, the second after their implementation

These user statistics were evaluated for the following periods: (a) between the RADUCATION launch in May 2022 to May 2023; (b) for the first six months after May 2023 when board exam questions on theoretical knowledge were first implemented; (c) for the six months after December 2023 when image-based board exam questions were first implemented; and (d) during the last months of use between the launch of the CMS in March 2024 to December 2024. The analyzed periods are displayed in Fig. 1.

### User experience and preferences

To evaluate the user experience and preferences, two surveys were conducted before and after implementing the image-based board exam questions in December 2023. For these surveys, links to the questionnaires were distributed via the FJR newsletter, the FJR Instagram channel, were directly accessible on the RADUCATION homepage, and via QR codes during the annual national congress of the German Roentgen Society in 2023 and 2024. The first survey was online from March 2023 to January 2024, and the second survey from June to August 2024, both on SurveyMonkey.

Both questionnaires included items about demographic information (age, gender, workplace, work position, and work experience), user-friendliness, preferred learning content (media type, content extent, preferred content language, and free vs payable content), self-assessment of usage (purpose and frequency), and the perceived value of RADUCATION. In the second survey, items were additionally included about the perceived value of board exam questions, the preferred type of exam questions (theoretical and image-based), and the quality of questions/solutions. Skipping of survey items was allowed to ensure higher compliance. If not all survey participants responded to a question, the absolute number of respondents is given in the “Results” section. If the results between the two surveys did not vary, they were reported as one. Differences between responses in survey 1 and 2 are stated separately. Both questionnaires are provided in the Supplemental Material 1 and 2.

### Statistical analyses

Descriptive statistics are provided with absolute and relative frequencies for categorical variables, the number of missing values, mean and standard deviation (SD), median and interquartile range, and minimum and maximum for continuous variables. We tested for gender differences in age groups, professional positions, and between the cohorts of both studies by performing a multivariable logistic regression with gender as the outcome variable and age groups, professional positions, and cohorts as predictor variables. The association of different variables for user evaluation with the predictor variables gender, age, position, survey year, training year, and working place (e.g., university vs regular hospital) was examined using multivariable logistic regression models separately for each user evaluation variable as outcome. User experience variables with a range of answers (e.g., very good, good, neutral, bad, very bad) were dichotomized to allow for more robust (higher sample size) and easier to interpret analyses. Odds ratios (OR) are provided with 95% confidence intervals (CIs). All subjects with available cases were included in the regression models. All statistical calculations were performed in R version 4.4.1.

## Results

**Fig. 2:**
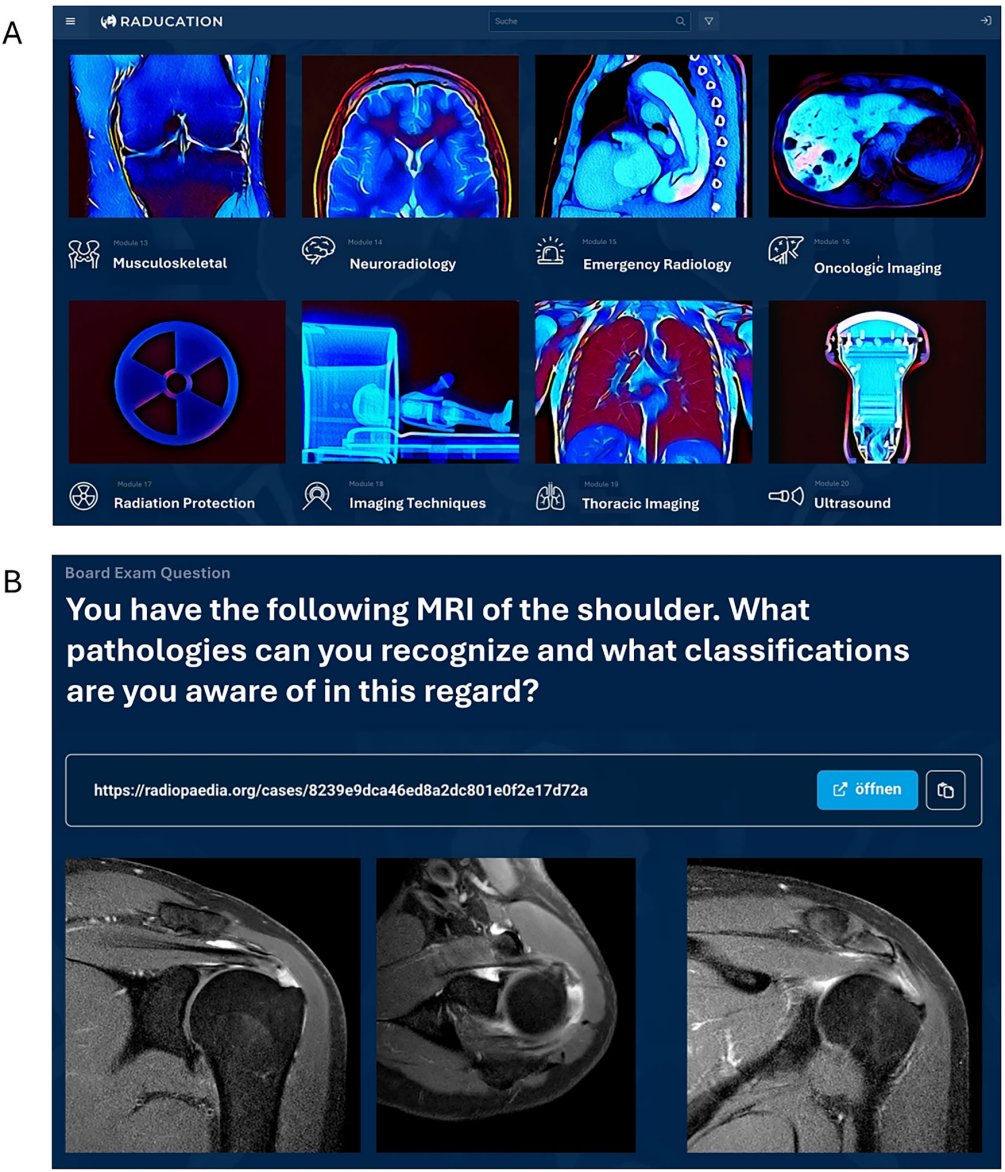
Graphical Impression of the RADUCATION starting page (**A**) and a board exam question (**B**). Images for each module (**A**) were created based on radiological images or radiographs of objects by a volunteer of the German Young Radiology Forum (author M. Kolb). As DICOM viewers for image stacks are still under implementation, image material fitting to the protocolized board exam questions (**B**) is selected from Radiopaedia, and a link to cases with hidden diagnoses from radiopaedia is provided in addition to the corresponding images. After reading the images, users can click on a response button to get the correct answer, including the corresponding learning objectives from the GTC and learning content associated with the specific learning objective. A translation was conducted for exemplary display; the RADUCATION homepage is designed in German

Graphical impressions of the RADUCATION platform are provided in Fig. 2.

### User statistics

After the launch of the RADUCATION homepage and before the implementation of board exam questions (May 2022 to May 2023), RADUCATION had 4468 active users (average of 372 per month) with a mean interaction time of 3.09 min. Most users accessed RADUCATION directly (*n* = 827), while a minority found the webpage via search engines (organic search, *n* = 179) or social media (organic social, *n* = 189). Concerning subparts of the webpage, most accesses were measured for the GTC (*n* = 5919), the abdominal imaging module (*n* = 2765), the login page (*n* = 2706), and the module on imaging techniques (*n* = 967).

In the first six months after the implementation of board exam questions on theoretical knowledge (May–October 2023), there were *n* = 2746 active users (average of 458 per month) with a mean interaction time of 3.53 min. The most commonly accessed part of the webpage was the GTC (*n* = 1758), followed by the newly implemented board exam questions (*n* = 1445), the abdominal imaging module (*n* = 1015), the login page (*n* = 686), and a list with learning content for board exam preparation (*n* = 513). The module on imaging techniques was the seventh most accessed part (*n* = 321).

Six months after additionally implementing image-based board exam questions (December 2023–May 2024), *n* = 3679 active users were registered (average of 613 per month) with a mean interaction time of 5.58 min. Board exam questions were now the most frequently used part of the website (*n* = 4122), followed by the GTC (*n* = 2156), the login page (*n* = 1108), the abdominal imaging module (*n* = 11,035), the content list for board exam preparation (*n* = 476), and the module on imaging techniques (*n* = 344).

During the last period before manuscript preparation, from March to December 2024, according to the CMS system, *n* = 15,828 users (average of 1583 per month) from Germany, Switzerland, and Austria were registered. The board exam question part of the webpage remained the most popular, accessed by *n* = 2850 users with a total number of 10,832 accesses. During this last year, most users accessed the RADUCATION webpage via Google search (*n* = 11,161), followed by referral from the FJR homepage (*n* = 564). About 80,000 interactions with the webpage were registered via computers, and 47,000 via mobile devices.

**Fig. 3:**
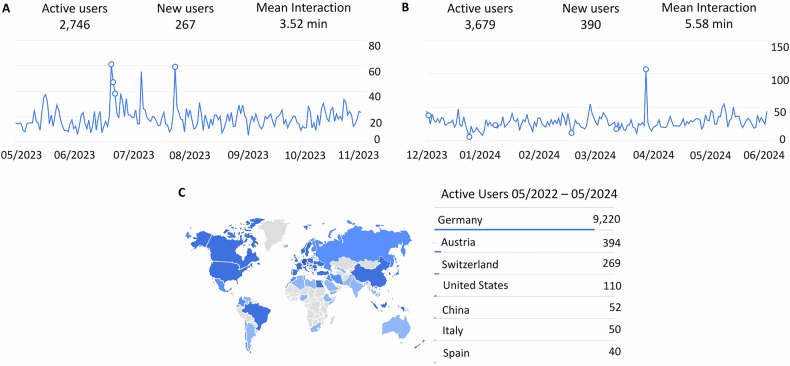
RADUCATION user numbers, mean interaction time, and user origin. An increase in user numbers and mean interaction time is shown from the first six months after the implementation of theoretical board exam questions (**A**) to the six months after the implementation of image-based board exam questions (**B**). Spikes in user numbers correspond to events such as the German Radiology Congress at which RADUCATION was advertised or to weekends at which German Young Radiology Forum volunteers worked on the RADUCATION platform. Most users accessed RADUCATION from German-speaking countries (**C**)

An overview of the user statistics is provided in Fig. 3.

### Survey collective

The 2023 survey had 121 participants, and the 2024 survey had 122 participants. An overview of demographic information is provided in Table 1. Most participants were female (2023: 64.1%, 2024: 62.3%). The largest group was made up of residents (69.9%), followed by board-certified radiologists (14.2%), attendings (8.8%), and others (e.g., students, chief physicians) (7.1%). The only gender difference was found for residents (OR women vs. men 1.24 (95% CI: 1.03–1.5)). Most participants worked in non-university hospitals (39.8%), followed by university hospitals (36.1%), private practice (16.6%), or other (7.5%). On average, participants were in their fourth training year (median year: 4.00 (3.00, 5.00)). Participants learned about RADUCATION from advertisements (63.4%, 154/243), followed by recommendations with 12.8% (31/243). In the 2024 survey, additionally, 13.9% (17/122) of participants had gotten to know RADUCATION by presentations of FJR volunteers in their departments.

**Table 1:** Participants’ demographics

		Total	Survey year	
Variable	*N*	*N* = 243^a^	2023 *N* = 121^a^	2024 *N* = 122^a^
Age	241			
< 26 years		12/241 (5.0%)	7/120 (5.8%)	5/121 (4.1%)
26–30 years		57/241 (23.7%)	26/120 (21.7%)	31/121 (25.6%)
31–35 years		83/241 (34.4%)	42/120 (35.0%)	41/121 (33.9%)
36–40 years		50/241 (20.7%)	24/120 (20.0%)	26/121 (21.5%)
41–45 years		15/241 (6.2%)	5/120 (4.2%)	10/121 (8.3%)
> 45 years		24/241 (10.0%)	16/120 (13.3%)	8/121 (6.6%)
Gender	239			
Diverse		2/239 (0.8%)	1/117 (0.9%)	1/122 (0.8%)
Male		86/239 (36.0%)	41/117 (35.0%)	45/122 (36.9%)
Female		151/239 (63.2%)	75/117 (64.1%)	76/122 (62.3%)
Position	239			
Resident		167/239 (69.9%)	79/118 (66.9%)	88/121 (72.7%)
Board-certified radiologist		34/239 (14.2%)	17/118 (14.4%)	17/121 (14.0%)
Attending		21/239 (8.8%)	11/118 (9.3%)	10/121 (8.3%)
Other		17/239 (7.1%)	11/118 (9.3%)	6/121 (5.0%)
Workplace	241			
Private practice		40/241 (16.6%)	21/119 (17.6%)	19/122 (15.6%)
Other		18/241 (7.5%)	14/119 (11.8%)	4/122 (3.3%)
Non-university hospital		96/241 (39.8%)	46/119 (38.7%)	50/122 (41.0%)
University hospital		87/241 (36.1%)	38/119 (31.9%)	49/122 (40.2%)
Year of training	160			
*N* missing		83	43	40
Mean (SD)		3.7 (1.4)	3.8 (1.4)	3.6 (1.5)
Median (Q1, Q3)		4.0 (3.0, 5.0)	4.0 (3.0, 5.0)	4.0 (2.0, 5.0)
Work experience	26			
< 5 years		16/26 (61.5%)	0/0 (NA%)	16/26 (61.5%)
5–10 years		8/26 (30.8%)	0/0 (NA%)	8/26 (30.8%)
> 10 years		2/26 (7.7%)	0/0 (NA%)	2/26 (7.7%)

^a^ *n*/*N* (%)

*Q1* first quartile, *Q3* third quartile, *SD* standard deviation

### User experience

**Fig. 4:**
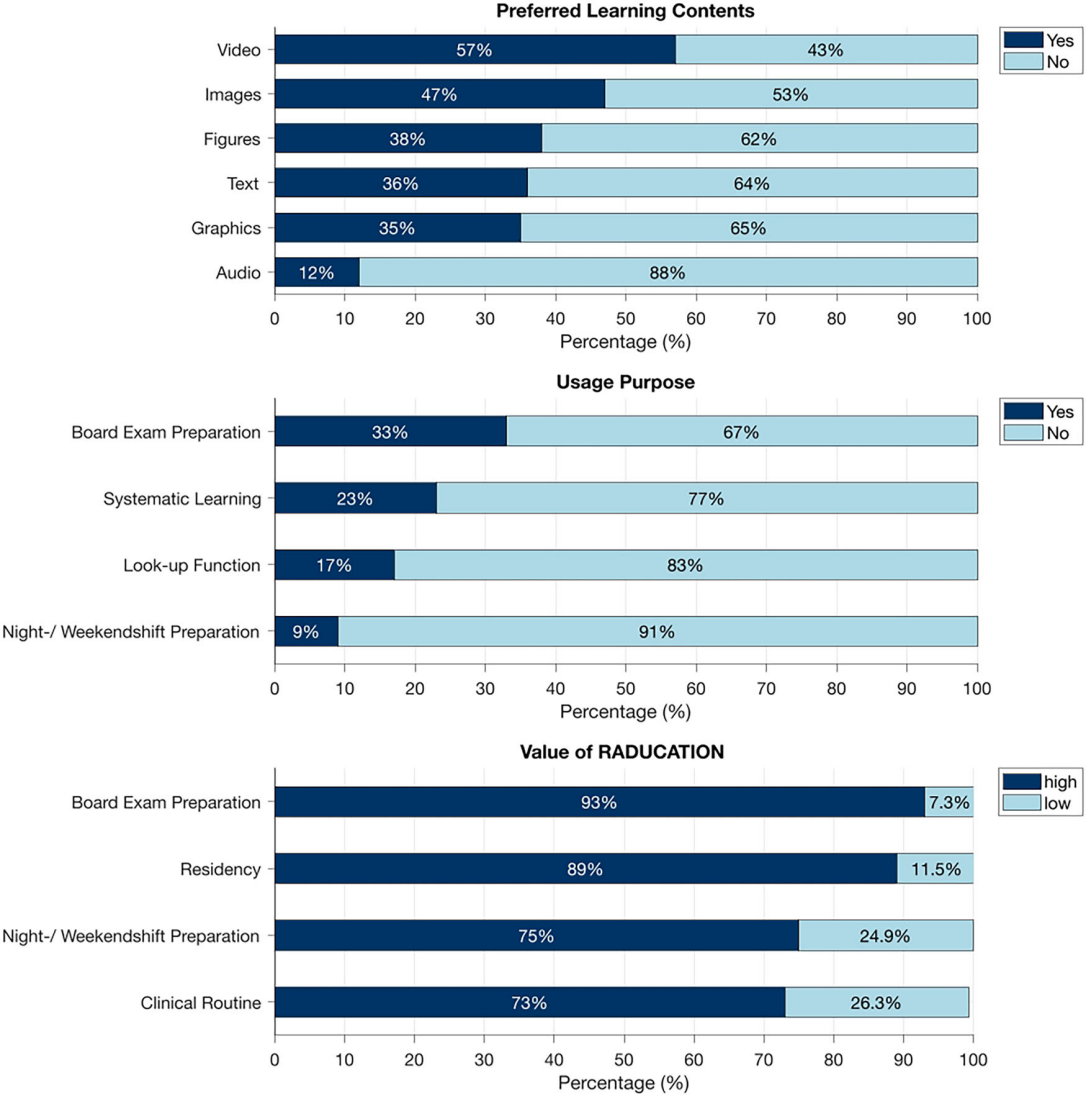
User preferences on media type for learning contents, usage purpose of RADUCATION, and subjectively perceived value of RADUCATION. The preferred media type of learning content was videos. Audio, such as podcasts, was the least attractive media type. RADUCATION was mainly used for board exam preparation. However, its value was also considered high for the residency in general, shift preparation, and clinical routine

The value of RADUCATION for preparation of board exams was judged as high or very high by 88.6% (85/96) of the participants of both surveys (Fig. 4). The value of RADUCATION for preparation of weekend and night shifts was deemed high or very high by 75.6% (62/82). For clinical routine, 73.4% found RADUCATION to be of high or very high value (69/94) (Fig. 4). Residents in their 4th and 5th year were more likely to use RADUCATION for exam preparation than residents in their 1st to 3rd year (OR 1.35 (95% CI: 1.15–1.59). In contrast, the value for preparation for weekend- and nightshifts was less critical for 4th- and 5th-year residents (OR 0.77 (95% CI: 0.59–0.99)).

Overall, RADUCATION was deemed user-friendly or very user-friendly by 86.0% (148/172). The layout of RADUCATION was judged as clear or very clear by 88.0% (154/175). Most survey participants stated using RADUCATION once per month (35.2%, 37/105) or once weekly (36.2%, 38/105).

### User preferences

Preferences concerning media type of learning contents and preferred use of RADUCATION are displayed in Fig. 4. When asked to choose their preferred media types for learning content (multiple answers allowed), the most frequently chosen preferred learning content was videos (57.2%), followed by image-based content (47.3%), and schematic illustrations (38.3%). Text-based content was preferred by 35.8%. The lowest preferences were found for audio media, e.g., podcasts, with 11.9%. Female participants were less likely than males to prefer illustrations (OR 0.78 (95% CI: 0.68; 0.88)) or audio content (OR 0.9 (95% CI: 0.83–0.99)), and more likely to use RADUCATION to look up information (OR 1.11 (95% CI: 1–1.23)). For all other learning contents or items, no gender differences were observed.

RADUCATION offers a filter function to exclude learning content in English, but most participants (81.1%, 99/122) stated they were using English content. Only 36.1% of participants (44/122) wished for more learning content, 34.4% for more board exam questions (42/122), and 19.7% (24/122) for more learning lists.

## Discussion

In this study, we evaluated the user statistics, user experience, and user preferences of the RADUCATION web platform for post-graduate radiological training with an emphasis on the impact of the implementation of board exam questions.

Based on surveys before and after board exam question implementation, we demonstrated that RADUCATION was consistently considered highly valuable for board exam preparation, especially by senior residents, and for preparation of night- and weekend shifts, mainly by younger residents. Survey participants preferred video and image-based learning contents and deemed RADUCATION user-friendly.

The webpage user statistics showed continuously increasing user numbers and interaction times. After the implementation of board exam questions derived from real exam protocols, these quickly became the most popular part of the webpage. Also, information on imaging techniques was frequently accessed.

Concerning the comparability of our results, there are only a few digital learning platforms for postgraduate medical training with available scientific evaluations. The KOLEGEA platform was developed for general practitioners in Germany and, in a survey among users, was perceived as a helpful supplement [5]. Notably, the presented patient cases were highly valued, as was the opportunity for professional exchange [5]. The practical interaction with patient cases can be compared to that with the image-based board exam questions in RADUCATION. However, professional exchange is only indirectly given in the RADUCATION platform by contact options to the FJR volunteers. In contrast to the KOLEGEA platform, RADUCATION is structured according to a nationwide and by the German radiological societies agreed-upon curriculum. Another post-graduate educational platform from the literature provided weekly training sessions via Zoom for pathology training in Africa [6]. This demonstrates one of the major benefits of all digital teaching platforms: the accessibility independent of local infrastructure. While there are no life interactions on RADUCATION, it allows asynchronous learning and could be implemented into flipped classroom concepts, with, e.g., local clinical training sessions. Specifically in Radiology, numerous platforms offer case-based learning, such as the Conrad platform of the German Roentgen Society [7], the Education on Demand platform of the European Radiological Society (ESR) [8], or RSNA EdCentral. We did, however, not find a comparable concept with free-of-charge learning contents and board exam preparation based on original exam materials, structured to and with complete coverage of a previously consensually developed curriculum. Prospectively, other societies such as the ESR that already offer structured curricula and structured board exams such as the European Diploma in Radiology, could use RADUCATION as a model for their educational platforms.

It was surprising to note that the majority of participants did not request further learning content or board exam questions. This not only provides valuable information concerning the further development of the RADUCATION platform towards increasing the quality instead of quantity, but can also be supported by the Cognitive Load Theory [9]. According to the Cognitive Load Theory, while there is an unlimited long-term memory, working memory is limited. Therefore, schemas are constructed in the long-term memory to handle high amounts of information while learning something [9]. In medicine, especially in radiology as a large interdisciplinary specialty, it is thus more important to ensure schema recognition than to provide large amounts of details. Indeed, RADUCATION already supports constructing schemas as all learning content and board exam questions are structured according to a fixed set of learning objectives of the GTC. With each read of a learning content or a board exam question, the trainee is referred to the respective learning objective. Theoretical knowledge and practical capability of image-based pathology recognition are thus trained under the schema of, e.g., a certain disease context as defined in a learning objective. According to the participants’ feedback, the amount of learning material on RADUCATION is sufficient. In line with the theoretical framework of the Cognitive Load Theory, more learning material would likely lead to an unnecessary mental overload. While excessive content can cause mental overload and board exam questions have become the most popular part of RADUCATION, it nevertheless remains essential to offer a comprehensive curriculum covering all radiological training. Moreover, focusing solely on external performance metrics, such as exam question accuracy, risks shifting intrinsic motivation toward the external reward. However, if the reward—in our case, the correct answer—is linked to the performance level, as is the case with exam questions, this effect is less likely to occur.

Interestingly, most survey participants were from non-university hospitals. This indicates that, although most FJR volunteers who created the GTC and RADUCATION are from university hospitals, the platform also became known amongst colleagues from non-university hospitals. Indeed, it might address a unique need in non-university hospitals, where colleagues have less access to congresses or expensive description-based literature and educational sessions. This also demonstrates the successful distribution of RADUCATION as a learning method embedded in a Community of Practice, from which learners nationwide can benefit equally [10]. Prospectively, it should be evaluated how RADUCATION can further advance using artificial intelligence (AI) tools such as natural language models beyond the already implemented natural language search function. This might, e.g., include an oral exam simulation using an interactive chatbot such as ChatGPT. However, compared to AI, it is the special value of training platforms such as RADUCATION to provide quality-assured, peer-developed educational content, which will remain indispensable to maintain reliability and alignment with professional standards.

### Limitations

This study has several limitations, including that the user statistics could only be collected upon consent, and internet bots could not be differentiated from human users in webpage statistics. Thus, actual user numbers could be higher due to users who did not accept cookies or lower due to registered accesses by bots. However, our data seems plausible compared to interaction data from other webpages, and bot accesses can be assumed to be constant over time.

As inherent to surveys, we cannot rule out a selection bias, with participants being particularly familiar with RADUCATION or deeming its value high, being more likely to participate, potentially leading to an overestimation of satisfaction and perceived usefulness. This is likely also why we observed a higher percentage of female participants (63%) than those who passed the radiological board exams in Germany in 2023 (227 out of 560, 40.5%) [11]. The first survey assessed users’ perceptions before introducing image-based questions in December 2023. It was accessible between May 2023 and January 2024, with a short time interval at the end during which the image-based questions were already available. However, most survey participants were registered before December 2023. To ensure overall compliance, questions could be skipped. Thus, the number of respondents per question varies, which, however, has been transparently reported for each respective item in the “Results” section.

In conclusion, we demonstrated the high value and increased use of RADUCATION as a digital learning platform, structured to a nationwide competency-based curriculum for the radiology residency. RADUCATION can serve as a best practice example of a modern self-regulated learning environment created by peers for postgraduate medical training for international societies such as the ESR or other specialties worldwide. Including exam content into such learning platforms is advisable to ensure relevance and increase user interaction.

## Supplementary information

ELECTRONIC SUPPLEMENTARY MATERIAL

## Data Availability

All data are available from the corresponding author upon request.

## Abbreviations

AI: Artificial intelligence
CI: Confidence interval
CMS: Content management system
ESR: European Society of Radiology
FJR: German Young Radiology Forum (Forum Junge Radiologie)
GTC: German training curriculum
OR: Odds ratio
