# Supplementary material for: Longitudinal evaluation of RADUCATION: a digital learning environment for the radiology residency structured to a competency-based curriculum by the German Young Radiology Forum
Source: Insights Imaging. 2025 Dec 8;16:274. doi: 10.1186/s13244-025-02135-x (PMC12686237; doi:10.1186/s13244-025-02135-x)
Supplement: Supplementary file 1 — ELECTRONIC SUPPLEMENTARY MATERIAL [file 13244_2025_2135_MOESM1_ESM.pdf]

Longitudinal evaluation of RADUCATION: a digital learning environment for the radiological residency structured to a competency-based curriculum by the German Young Radiology Forum

ELECTRONIC SUPPLEMENTARY MATERIAL

Supplemental Material 1, RADUCATION Evaluation - Questionnaire 2023

Q1: Your age

a) 0-20
b) 20-25
c) 26-30
d) 31-35
e) 36-40
f) 41-45
g) 45

Q2: Your gender

a) Female
b) Male
c) Diverse
d) None of the above

Q3: Your workplace

a) University hospital
b) Non-university hospital
c) Private Practice (MVZ)
d) Other (please specify)

Q4: Your position

a) Student
b) Resident
c) Board-certified radiologist
d) Attending
e) Chief physician
f) Other (please specify)

Q5: Your year of radiological training

a) First
b) Second
c) Third
d) Fourth
e) Fifth
f) Other (please specify)

Q6: How did you become aware of RADUCATION?

a) Advertisement by the German Roentgen Society / German Young Radiology Forum
b) Recommendation
c) Internet search
d) Other (please specify)

Q7: How clear do you find the structure of RADUCATION?

a) Very clear
b) Clear
c) Don’t know
d) Less clear
e) Not clear

Q8: How do you rate the user-friendliness/navigation of the platform?

a) Very good
b) Good
c) Don’t know
d) Rather poor
e) Poor

Q9: What type of learning content do you prefer?

a) Text
b) Video
c) Graphics
d) Schematic illustrations
e) Images
f) Audio
g) Other (please specify)

Q10: Have you already used RADUCATION for learning?

a) Yes
b) No

Q11: How often do you use RADUCATION?

a) Multiple times per week
b) Once per week
c) Once per month
d) Less than once per month
e) Never
f) Other (please specify)

Q12: For what purpose do you use RADUCATION?

a) Board exam preparation
b) Preparation of first night-/weekend shifts
c) Reference tool
d) Systematic learning
e) Structuring of learning content
f) Other (please specify)

Q13: How do you rate the value of RADUCATION for board exam preparation?

a) Very high
b) High
c) Don’t know
d) Less high
e) Not high

Q14: How do you rate the value of RADUCATION for preparing yourself for night-/weekend shifts?

a) Very high
b) High
c) Don’t know
d) Less high
e) Not high

Q15: How do you rate the value of RADUCATION for clinical routine?

a) Very high
b) High
c) Don’t know
d) Less high
e) Not high

Q16: Do you use paid learning content besides eREF?

a) Yes
b) No

Q17: Do you use English learning content?

a) Yes
b) No

Q18: How do you assess the learning content on RADUCATION in terms of:

…Scope?
…Clarity?
…Accuracy?
…Relevance?
…Timeliness?

a) Very good
b) Good
c) Neutral
d) Rather poor
e) Poor

Q19: External providers would create new learning content for RADUCATION (e.g., lectures on specific learning objectives) and provide it for free if their company logos were displayed on the presentation slides. Would you support such collaborations?

a) Yes, absolutely
b) Rather yes
c) Don’t know
d) Rather no
e) No, absolutely not

Q20: Has RADUCATION sparked your interest in the German Young Radiology Forum?

a) Yes, absolutely
b) Rather yes
c) Don’t know
d) Rather no
e) No, absolutely not

Q21: Would you recommend RADUCATION to a colleague?

a) Yes, absolutely
b) Rather yes
c) Don’t know
d) Rather no
e) No, absolutely not

Supplemental Material 2, RADUCATION Evaluation – Questionnaire 2024

Q1: Your Age

a) < 20 years
b) 20 – 25 years
c) 26 – 30 years
d) 31 – 35 years
e) 36 – 40 years
f) 41 – 45 years
g) 45 years

Q2: Your Gender

e) Female
f) Male
g) Diverse
h) None of the above

Q3: Your Workplace

e) University hospital
f) Non-university hospital
g) Private Practice (MVZ)
h) Other (please specify)

Q4: Your Position

g) Student
h) Resident
i) Board-certified radiologist
j) Attending
k) Chief physician
l) Other (please specify)

Q5: If you are a student, which semester are you in?

Q6: If you are a resident doctor: in which year of training, are you?

a) First
b) Second
c) Third
d) Fourth
e) Fifth
f) Other (please specify)

Q7: If you are a board-certified radiologist, attending, or chief physician: How many years of experience do you have since your board exams?

a) < 5 years
b) 5 – 10 years
c) 10 – 15 years
d) 15 – 20 years
e) 20 – 30 years
f) 30 years

Q8: How did you become aware of RADUCATION?

e) Advertisement by the German Roentgen Society / German Young Radiology Forum
f) Presentation in the department
g) Recommendation
h) Internet search
i) Other (please specify)

Q9: How clear do you find the structure of RADUCATION?

a) Very clear
b) Clear
c) Don’t know
d) Less clear
e) Not clear

Q10: How do you rate the user-friendliness/navigation of the platform?

a) Very good
b) Good
c) Don’t know
d) Rather poor
e) Poor

Q11: What type of learning content do you prefer? (Select your two favorites)

h) Text
i) Video
j) Graphics
k) Schematic illustrations
l) Images
m) Audio
n) Other (please specify)

Q12: Have you already used RADUCATION for learning?

a) Yes
b) No

Q13: How often do you use RADUCATION?

a) Multiple times per week
b) Once per week
c) Once per month
d) Less than once per month
e) Never
f) Other ________________

Q14: For what purposes do you use RADUCATION? (Multiple selections possible)

g) Board exam preparation
h) Preparation of first night-/weekend shifts
i) Reference tool
j) Systematic learning
k) Structuring of learning content
l) Other (please specify)

Q15: How do you rate the value of RADUCATION for board exam preparation?

a) Very high
b) High
c) Don’t know
d) Less high
e) Not high

Q16: How do you rate the value of RADUCATION for preparing yourself for night-/weekend shifts?

a) Very high
b) High
c) Don’t know
d) Less high
e) Not high

Q17: How do you rate the value of RADUCATION for clinical routine?

a) Very high
b) High
c) Don’t know
d) Less high
e) Not high

Q18: How do you rate the value of the exam questions for board exam preparation?

a) Very high
b) High
c) Don't know
d) Less high
e) Not high

Q19: What do you find more useful for board exam preparation? (Multiple selections possible)

a) Image-based questions (e.g., description and interpretation of CT, MRI images)
b) Text-based questions (e.g., technical questions, definitions, classifications, differential diagnoses)
c) Other question formats

Q20: What is your opinion on eREF? (Multiple selections possible)

a) I would like more eREF content
b) I would like less eREF content
c) My free eREF access via the German Young Radiology Forum has expired
d) I do not have access

Q21: Do you use paid learning content besides eREF?

a) Yes
b) No

Q22: Do you use English-language content?

a) Yes
b) No

Q23: How do you assess the learning content on RADUCATION regarding:

…Scope?
…Clarity?
…Accuracy?
…Relevance?
…Timeliness?

a) Very good
b) Good
c) Neutral
d) Rather poor
e) Poor

Q24: How do you rate the answers to the board exam questions created by the German Young Radiology Forum based on exam protocol answers regarding:

…Scope?
…Clarity?
…Accuracy?
…Relevance?
…Timeliness?

a) Very good
b) Good
c) Neutral
d) Rather poor
e) Poor

Q25: What would you like more of?

a) Learning content
b) Board exam questions
c) Learning lists
d) Other (please specify)

Q26: External providers would create new content for RADUCATION (e.g., lectures on specific learning objectives) and provide it for free if their company logos were displayed on the presentation slides. Would you support such collaborations?

a) Yes, absolutely
b) Rather yes
c) Don't know
d) Rather no
e) No, absolutely not

Q27: Has RADUCATION sparked your interest in the German Young Radiology Forum?

a) Yes, absolutely
b) Rather yes
c) Don't know
d) Rather no
e) No, absolutely not

Q28: Would you recommend RADUCATION to a colleague?

a) Yes, absolutely
b) Rather yes
c) Don't know
d) Rather no
e) No, absolutely not

Q29: What is missing from RADUCATION, and what can we improve?
